# Researches on Stem and Progenitor Cells in Intervertebral Discs: An Analysis of the Scientific Landscape

**DOI:** 10.1155/2022/1274580

**Published:** 2022-09-01

**Authors:** Yunzhong Cheng, Honghao Yang, Yong Hai, Yuzeng Liu

**Affiliations:** Department of Orthopedic Surgery, Beijing Chao-Yang Hospital, Capital Medical University, Beijing 100020, China

## Abstract

Low back pain (LBP) is a common clinical symptom, and the prevalence is ranged from 60% to 70%. With the deepening of basic research, the development of intervertebral disc regeneration-oriented cell therapy, especially stem and progenitor cells therapy, showed good research prospects and was expected to become new methods of treatment for LBP. Our study is aimed at analyzing the scientific output of stem and progenitor cells in intervertebral discs and at driving future research into new publications. Researches focused on this file were searched from the Science Citation Index Expanded (SCI-E) of the Web of Science (WOS) core collection database and were screened according to inclusion criteria. We evaluated and visualized the results, including annual publications, citations, authors, organizations, countries, research directions, funds, and journals by bibliometric website, VOSviewer, and Citespace softwares on May 27, 2022. A total of 450 original articles and reviews were included, and the overall trend of the number of publications rapidly increased. In worldwide, China and the USA were the leading countries for research production. The retrieved 450 publications received 14322 citations, with an average of 31.83 citations and an H-index of 62. The most high-yield author, organization, country, research directions, funds, and journals were Chen QX from Zhejiang University, Zhejiang University, China, Cell Biology, National Natural Science Foundation of China, and Spine, respectively. Keywords cluster analysis showed the research hotspots in the future, including “human intervertebral disc”, “adipose-derived mesenchymal stem cell”, “intervertebral disc degeneration”, “degenerative disc model”, “nucleus pulposus regeneration”, “human cartilage”, “3d culture”, “shrinkage-free preparation”, and “polylactide disc”. Furthermore, with accumulating evidence demonstrating the role of stem and progenitor cells in intervertebral discs, “microenvironment”, “activation”, “intervertebral disc degeneration”, and “oxidative stress” are becoming the research frontiers and trends.

## 1. Introduction

The intervertebral disc consists of the nucleus pulposus, annulus fibrosus, and cartilage endplates. Proteoglycan in the nucleus pulposus has a large amount of anions and high osmotic pressure, which can absorb water and swell. The expansion converts the compressive load into a tensile effect on the annulus, which acts as a tension “skin” to limit the expansion of the nucleus pulposus [1]. Due to the structural characteristics of the fibrous annulus, its resistance to compression is far less than that of tensile capacity. The fibrous ring structure is easily destroyed when a large compressive load occurred [2]. More importantly, the intervertebral disc itself lacks blood supply; it is very difficult to self-healing once degeneration and damage happened [3]. In recent years, researches on the pathophysiology of intervertebral disc degeneration have opened a new avenue for disc regeneration therapy [4], particularly stem and progenitor cells therapy for intervertebral disc problems.

Bibliometric analysis and visualization are not only more effective methods to assess the thematic development of structural contents. More importantly, it can help researchers to better understand comprehensively about hotspots, frontiers, and trends in particular topic [5–7]. Science Citation Index Expanded (SCI-E) of Web of Science (WOS) Core Collection Database is widely used as an important tool for scientific statistics and scientific evaluation [8]. Thanks to the quantitative construction of this database and the qualitative contribution of the bibliometrics, most cited publications, top high-yield countries, organizations, authors, research directions, and funds, as well as journals, can be comprehensively analyzed.

However, no bibliometric literature on stem and progenitor cells in intervertebral discs has been researched and reported. Our study aims to draw the outline of the intellectual connections within the dynamic changing of scientific knowledge in the field of stem and progenitor cells in intervertebral discs by making good use of the citation database (SCI-E) and the software tools (https://bibliometric.com/, VOSviewer, and Citespace). These results can benefit scholars by better understanding future research directions and trends.

## 2. Method

### 2.1. Data Collecting

The literature data were retrieved through the SCI-E of WOS Core Collection Database in Capital Medical University Library. The search query was“((TI=(Stem Cell OR Progenitor Cell) AND TI=(nucleus pulposus OR disc OR intervertebral discs OR annulus fibrosis OR endplates OR perichondrium)) AND LA=(English) AND DT=(Article or review)”. The literature searching was accomplished within a single day to avoid the bias due to database updates on May 27, 2022. The records were exported by “full records and cited references” in plain text file format and tab delimited file format, respectively.

### 2.2. Bibliometric Analysis

The trends of publications and citations were charted annually. Contribution of all countries by publications was made by a pie chart. A total number of publications and sum of total citations from 1999 to 2022 were obtained. Top 20 Most Cited Articles were recorded and analyzed, including first author, article title, journals of publication, year of publication, total number of citations, and the impact factor of journals. The top 5 records, H-index, total citations, and average citations in terms of authors, organizations, and countries were tabulated directly. The top 5 research directions, funds, and journals with the most publications were meanwhile charted.

The co-authorship analysis of countries on stem and progenitor cells in intervertebral disc degeneration was analyzed by the bibliometric website (https://bibliometric.com/). The co-authorship relations in the analysis units of authors and organizations, the co-citation analysis of references, journals, and authors were all mapped by VOSviewer_-_1.6.11 software (Nees Jan van Eck and Ludo Waltman, 2019).

Co-citation timeline of references by keywords, keywords clusters on stem and progenitor cells in intervertebral discs, top 25 references with the strongest citation bursts, and top 14 keywords with the strongest citation bursts, as well as details of top 9 clusters, were visualized by CiteSpace_5.8. R3 edition (Chaomei Chen, 2003-2022). The time slicing was selected from January, 1999 to December, 2021. Years per slice was picked by one. The rest of the parameters are chosen by default setting. The reference was selected for co-citation timeline and burst analyses. The keyword was selected for burst analyses, and details of cluster with three different algorithms (LSI, LLR, MI) were exported into a table.

## 3. Results

### 3.1. General Information

**Figure 1:**
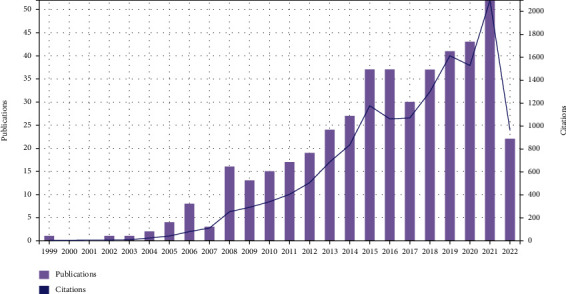
Annual publications and sum of times cited per year on stem and progenitor cells in intervertebral discs.

A total of 450 articles and reviews were retrieved in the SCI-E of WOS Core Collection Database, with a sum of 14322 times cited, average citations of 31.83 per item, and an H-index of 62.  Figure 1 showed the annual publications and sum of times cited per year on stem and progenitor cells in intervertebral discs. The year with most publication was 2021 (*n* = 52), and the number of publications showed a fluctuating increase year by year. In addition, the citation started in 2003 (*n* = 6), and the year with most times cited was 2021 (*n* = 2098), and citations increased linearly year by year.

### 3.2. Publications Distribution in Different Countries of the World

**Figure 2:**
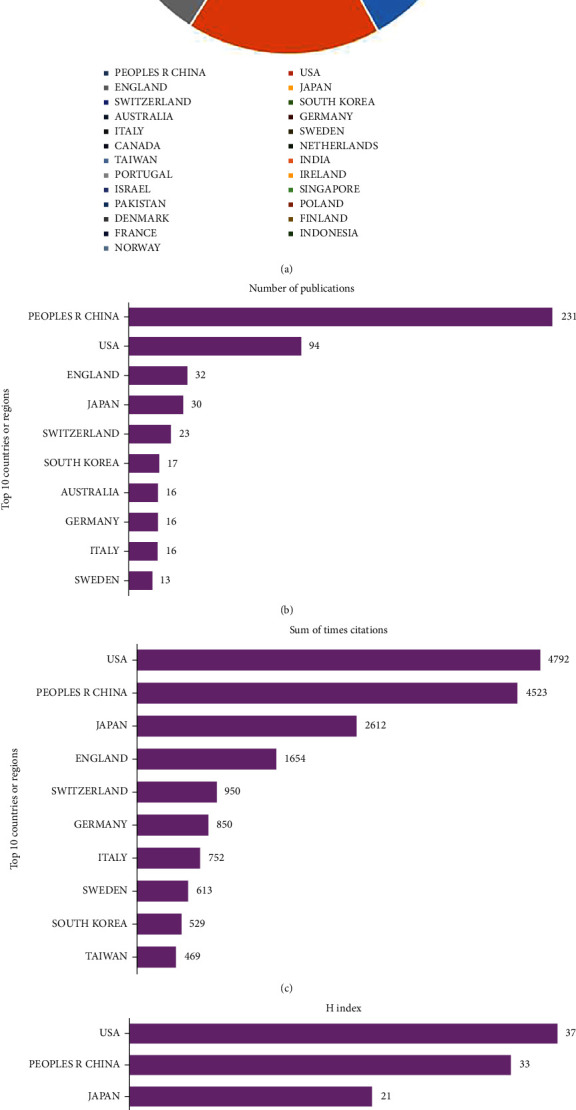
(a) Contribution of all countries by Publications. (b–d) Total number of publications, sum of total citations, and H-index of top 10 countries on stem and progenitor cells in intervertebral discs.

A total of 25 countries were retrieved with publications on stem and progenitor cells in intervertebral discs. China and the USA were in a dominant position, accounting for more than 70% in all over the world (Figure 2(a)). China had contributed 231 articles (51.33%) at the top. The USA is the second contributing country with 94 articles (20.89%), followed by England with 32 articles (7.11%), Japan with 30 articles (6.67%), and Switzerland both with 23 articles (5.11%) (Figure 2(b)). Total times citations of the USA were 4792 at the first, followed by China (4523), Japan (2612), England (1654), and Switzerland (950) (Figure 2(c)). Meanwhile, the H-index of the USA was 37 in the first place, China was the second with 33, Japan (21), England (18), and Switzerland (15) (Figure 2(d)).

### 3.3. Top 20 Most Cited Articles

A total of 450 articles from Web of Science were collected. Top 20 most cited articles on stem and progenitor cells in intervertebral discs are showed in Table 1, including first author, article title, journals of publication, year of publication, total number of citations, and the impact factor of journals. The total citations of the top 20 articles ranged from 141 to 311. The top article had 311 citations and was published in 2003 by Sakai D [9], followed by Sakai D [10] with 277 citations in 2006 and Sakai D with 267 citations in 2012 [11]. The oldest article was published by Arai F in 2002 [12], and the most recent article in top 20 was published in 2016 by Richardson SM [13]. More importantly, the impact factor of 1 article was more than 20, the impact factor of 6 articles was more than 14, and the impact factor of 10 articles was more than 6.

### 3.4. Contribution of Authors, Organizations, and Countries

1909 authors, 532 organizations, and 25 countries contributed to this field, respectively.  Table 2 showed that the top author with most publications was Chen QX (*n* = 22) from Zhejiang University and Zhou Y (*n* = 22) from Army Medical University [14, 15], followed by Li FC (*n* = 20) from Zhejiang University [16], Liang CZ (*n* = 20) from Zhejiang University [17], and Li H (*n* = 19) from Shanghai Jiao Tong University [18]. Of the 532 organizations, Zhejiang University, Army Medical University, League of European Research Universities-LERU, Huazhong University of Science & Technology, and University of Hong Kong had contributed 33, 29, 29, 26, and 18 publications, respectively. The top 5 countries with the most publications were China (*n* = 231), the USA (*n* = 94), England (*n* = 32), Japan (*n* = 30), and Switzerland (*n* = 23). What's more, the corresponding records, H-index, total citations of the top 5 authors, organizations, and countries were meanwhile showed in Table 2.

### 3.5. Contribution of Research Directions, Funds, and Journals

There were 38 research directions, 478 funds, and 167 Journals contributed to publications on stem and progenitor cells in intervertebral discs, respectively. Cell biology occupied the most records (*n* = 181), the highest H-index of 35, the highest total citations (*n* = 4421), and average citations (*n* = 24.43) [19]. Neurosciences and neurology occupied the most average citations (*n* = 52.94). Orthopedics had the second records (*n* = 92), the highest H-index of 35, the second total citations (*n* = 4347), and the second average citations (*n* = 47.25) [20]. In addition, National Natural Science Foundation of China had the most records (*n* = 157), the highest H-index of 27, the highest total citations (*n* = 2798), and average citations (*n* = 17.82) [21, 22]. National Institutes of Health (NIH), USA, and United States Department of Health & Human Services were the second with records (*n* = 30), H-index of 19, the total citations (*n* = 1239), and the highest average citations (*n* = 41.30). Furthermore, *Spine* occupied the most records (*n* = 27), the highest H-index of 20, the highest total citations (*n* = 2025), and the highest average citations (*n* = 75.00) [23, 24]. *Stem Cells International* was the second with records (*n* = 23), H-index of 12, the total citations (*n* = 359), and average citations (*n* = 15.61) [25–27]. Furthermore, the corresponding records, H-index, total citations, and average citations of the top 5 research directions, funds, and journals with the most publications were meanwhile list in Table 3.

### 3.6. Co-Authorship Analysis of Publications

**Figure 3:**
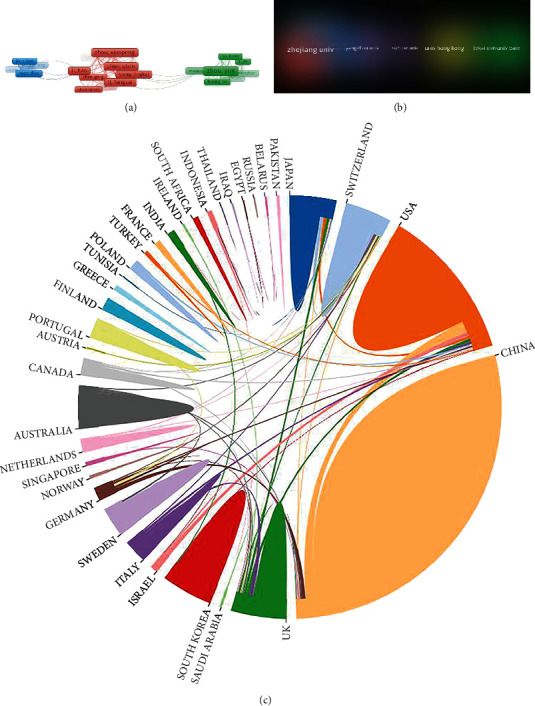
The co-authorship analysis of (a) authors, (b) organizations, and (c) countries on stem and progenitor cells in intervertebral discs. (The size of the frames represents the proportion of the author in the analysis. The larger the frames, the greater the contribution. The line between the frames represents the connection between the authors. The more or thicker the line, the stronger the connection. The color of the area where organization is located represents the connection between organizations. The darker the color, the closer the collaboration organization; the larger the area, the greater the contribution).

Zhou XP had the most co-authorship strength (total link strength = 87), with 17 documents and 348 citations [28], followed by Liang CZ (total link strength = 85) with 16 documents and 392 citations [29] and Li FC (total link strength = 84) with 16 documents and 358 citations [30] (Figure 3(a)). Moreover, the closest collaboration organization was Shanghai Jiao Tong University (total link strength = 20) with 15 documents and 290 citations, the second organization was Yangzhou University (total link strength = 18) with 13 documents and 224 citations, and the third was Chinese Orthopaedic Regenerative Medicine Society (total link strength = 14) with 6 documents and 239 citations [31–33] (Figure 3(b)). Besides, the strongest collaborative country was the USA (total link strength = 58) with 94 documents and 4791 citations, followed by China (total link strength = 35) with 231 documents and 4522 citations and Japan (total link strength = 24) with 30 documents and 2612 citations (Figure 3(c)).

### 3.7. Co-Citation Analysis of Publications

**Figure 4:**
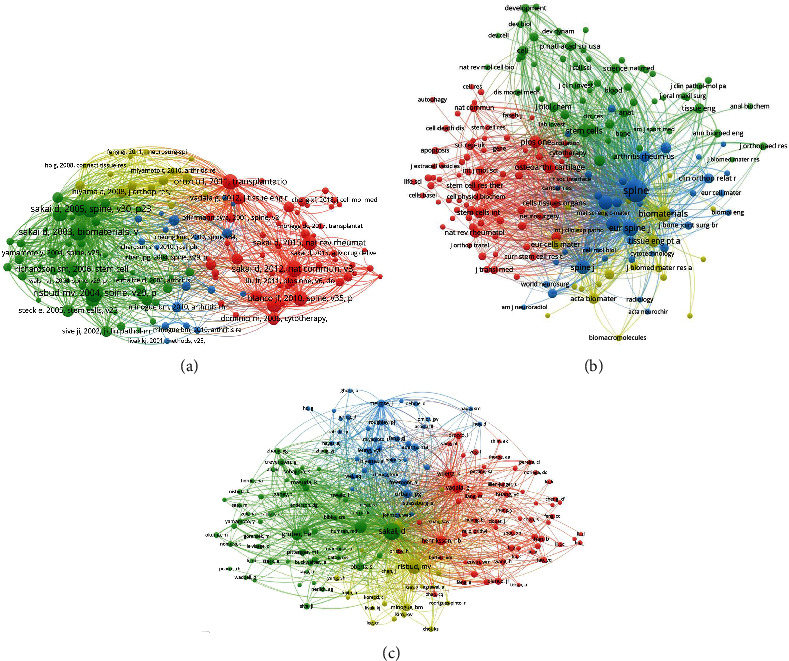
The co-citation analysis of (a) references, (b) journals, and (c) authors on stem and progenitor cells in intervertebral discs. (A point in the figure represents one reference, journal, and author, respectively. The color of the point represents different clusters; the size of the point represents the number of citations for each reference, journal, and author, respectively. The more the number, the larger the point. The connection between the two points represents two papers are jointly cited by another paper, and the length of the connection between the two points represents the correlation between two articles; the shorter the line, the stronger the correlation).

The most co-citation reference (*n* = 96) titled “Differentiation of mesenchymal stem cells towards a nucleus pulposus-like phenotype in vitro: implications for cell-based transplantation therapy” was published by Risbud MV on *Spine* in 2004 [34]. The second reference (*n* = 92) titled “Differentiation of mesenchymal stem cells transplanted to a rabbit degenerative disc model - Potential and limitations for stem cell therapy in disc regeneration” was published by Sakai D on *Spine* in 2005 [35], The third reference (*n* = 92) was “Transplantation of mesenchymal stem cells embedded in Atelocollagen((R)) gel to the intervertebral disc: a potential therapeutic model for disc degeneration”, published by Sakai D on *Biomaterials* in 2003 (Figure 4(a)). On the other hand, the most co-citation journal was *Spine* (*n* = 3179) [36], followed by *European Spine Journal* (*n* = 774) [37] and *Spine Journal* (*n* = 582) [38] (Figure 4(b)). Furthermore, the most co-citation author was Sakai D (*n* = 547) [9], the second was Risbud MV (*n* = 333) [34], and the third was Richardson SM (*n* = 234) [39] (Figure 4(c)).

### 3.8. Co-Citation Timeline of References and Burst Analysis

**Figure 5:**
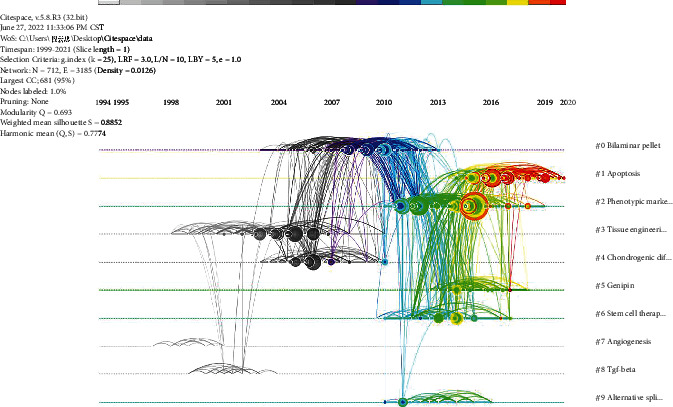
Co-citation timeline of references by keywords on stem and progenitor cells in intervertebral discs. (The nodes represent the references. The larger the node, the more citations the reference. The colors of the nodes from the inside to the outside correspond to color scale, which represents the total co-citations for the reference in the specific year. The line between two nodes represents two references co-citations. The thicker the line, the more the co-citations. The color of the connection line corresponds to the color mark above, which can reflect the time when two references were first co-cited.)

**Figure 6:**
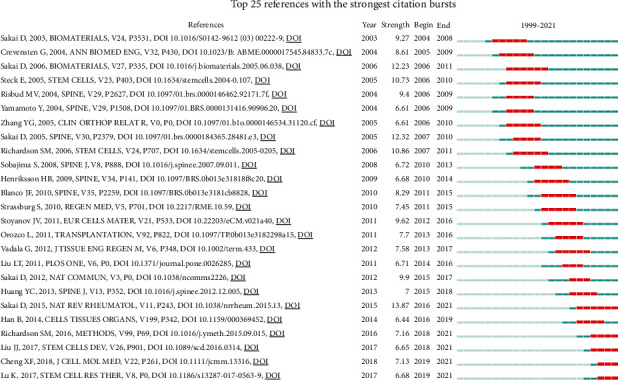
Top 25 references with the strongest citation bursts on stem and progenitor cells in intervertebral discs.

Co-citation of references for a timeline diagram was drawn by Citespace software (Figure 5). References to the same cluster are arranged on the timeline in chronological order of publication. “bilaminar pellet”, “apoptosis”, and “phenotypic markers” were the clusters with most published references. According to the year of publication, “tissue engineering”, “angiogenesis”, and “tgf-beta” were the clusters with the earliest references. “Apoptosis” and “phenotypic markers” were the clusters with the latest references. The top 25 references with the highest burst value are shown in Figure 6. The earliest reference with the strongest citation bursts was “Transplantation of mesenchymal stem cells embedded in Atelocollagen((R)) gel to the intervertebral disc: a potential therapeutic model for disc degeneration”, published by Sakai D on *Biomaterials* in 2003 [39]. The latest reference with the strongest citation bursts was “Mesenchymal stem cells deliver exogenous miR-21 via exosomes to inhibit nucleus pulposus cell apoptosis and reduce intervertebral disc degeneration”, published by Cheng XF on *Journal of Cellular and Molecular Medicine* in 2018 [18].

### 3.9. Keyword Visualization Analysis

**Figure 7:**
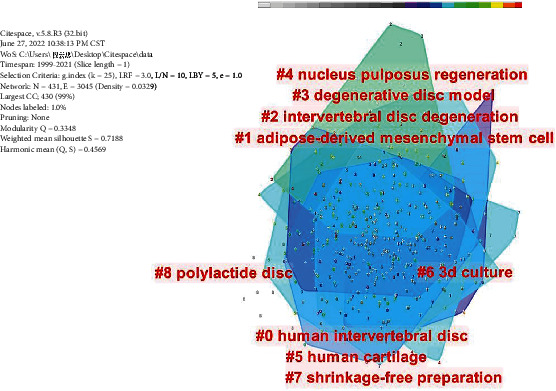
Keywords clusters on stem and progenitor cells in intervertebral discs. (A point in the figure represents a keyword. The color of the point represents different clusters, and the size of the point represents the co-occurrence for each keyword. The more the co-occurrence, the larger the point; # represents different cluster labels, #0 human intervertebral disc, #1 adipose-derived mesenchymal stem cell, #2 intervertebral disc degeneration, #3 degenerative disc model, #4 nucleus pulposus regeneration, #5 human cartilage, #6 3d culture, #7 shrinkage-free preparation, and #8 polylactide disc.)

The log-likelihood rate (LLR) algorithm was used to cluster all keywords by Citespace software, and the top 9 clusters are shown in Figure 7 and Table 4. Generally speaking, clustering module value (Q) > 0.3, indicating that the clustering structure is significant; the average contour value (S) > 0.7 means that the clustering is convincing. Q = 0.4569, and S = 0.7188 in our study. Each label was interconnected and developed, not independently exist. The color corresponding to the cluster area indicated the first time that a co-citation appeared. The clusters represented by green appeared later than the clusters represented by blue and purple. The smaller the cluster number stood for the more keywords the cluster contained. The cluster labels were as follows: #0 human intervertebral disc, #1 adipose-derived mesenchymal stem cell, #2 intervertebral disc degeneration, #3 degenerative disc model, #4 nucleus pulposus regeneration, #5 human cartilage, #6 3d culture, #7 shrinkage-free preparation, and #8 polylactide disc.

**Figure 8:**
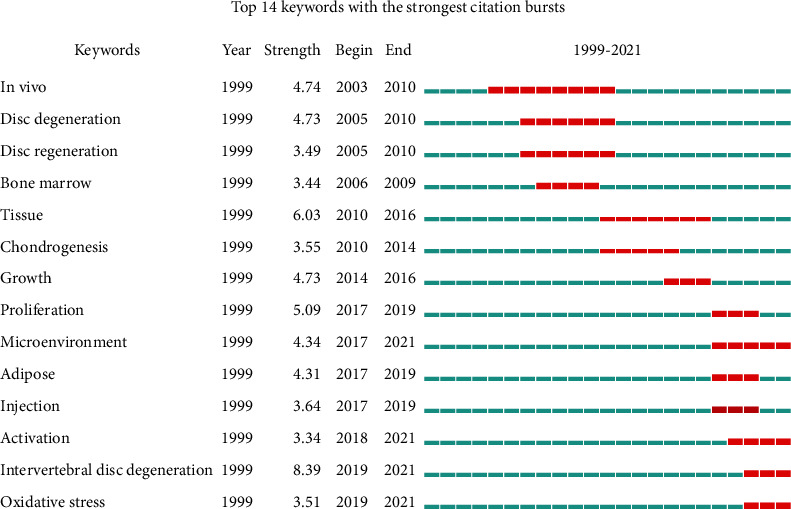
Top 14 keywords with the strongest citation bursts on stem and progenitor cells in intervertebral discs.

The top 14 keywords with the highest burst value are illustrated in Figure 8. The time period occupied by red on the right was the duration of the keywords. According to the burst strength and duration of the keywords, the transformation of domain research direction can be roughly divided into three stages. The first stage was from 2003 to 2009; the keywords were “in vivo”, “disc degeneration”, “disc regeneration”, and “bone marrow”. The second stage was from 2010 to 2016; the keywords were “tissue”, “chondrogenesis”, “growth”, and “proliferation”. The third stage was from 2017 to 2021; the keywords were “microenvironment”, “activation”, “intervertebral disc degeneration”, and “oxidative stress”.

## 4. Discussion

### 4.1. General Information and Bibliometric Analysis

The number of publications on a specific topic can reflect the popularity in this field. The researches regarding on stem and progenitor cells in intervertebral discs was initially published in 1999. The number of articles published increased rapidly from 2002 to 2022. On the other hand, the quality on a specific topic can be judged by the number of citations. There was a linear growth for the citation times from 1999 to 2022. From the result of Figure 1, we can know that the future trend on stem and progenitor cells in intervertebral discs looks very promising.

China was dominant in this field by the number of publications. Meanwhile, the publications of China and the USA accounted for 72.22%, which indicates a great contribution to this field by these two countries. It was might associated with large disc degeneration populations and high incidence in two countries [40]. China had the most publications. However, the USA had the highest citations and H-index, showing that China's research in this field was not deep enough. Sakai D, coming from Tokai University School of Medicine in Japan, has 5 most cited articles in the top 20 and 4 articles among them listed in top 4, with more than 265 total citations. He focused on mesenchymal stem cells embedded in Atelocollagen((R)) gel to the intervertebral disc and stem cell therapy for intervertebral disc regeneration [41].

From the analysis of number of publications issued by the author, the top 5 authors all came from China. Professor Chen QX from Zhejiang University in China has a great influence in this field. They had carried out numbers of researches on improving the biological repair function of nucleus pulposus mesenchymal stem cells by constructing biological scaffolds and introducing growth factors [42, 43]. Professor Zhou Yue from the Chinese Army Military Medical University was also made great contributions to the research direction of mesenchymal stem cell differentiation [42, 43].

Zhejiang University and Army Medical University were listed in the top 2, and only one institution was not in China in the top 5, which demonstrated that Chinese universities and institutions had a great contribution and influence in the field of stem and progenitor cells in intervertebral discs. From the analysis of number of publications issued by fund and journal, National Natural Science Foundation of China (NSFC) and National Institutes of Health (NIH), USA, were in the top 2, which was consistent with the greatest contribution of China and the USA. *Spine* and *Stem Cells International* were listed top 2 in all journals. The co-authorship analysis of authors, organizations, and countries on stem and progenitor cells in intervertebral discs showed the cooperation between them was not closely enough.

### 4.2. Research Hotspots on Stem and Progenitor Cells in Intervertebral Discs

Through the cluster analysis of keywords, we can clearly know the research hotspots on stem and progenitor cells in intervertebral discs.

#### 4.2.1. Cluster #0 Human Intervertebral Disc

The intervertebral disc is located between adjacent vertebral bodies and consists of the peripheral annulus fibrosus (annulus fibrosus, AF), central jelly-like nucleus pulposus (nucleus pulposus, NP), and cartilage endplate (cartilage endplate, CE). The peripheral AF is mainly composed of fibroblast-like annulus fibrosus. And the main cell types of NP are stellate chondroid cells. The bone endplate is a thin layer of cartilage, which is similar with articular cartilage tissue [44]. As all we know, human intervertebral discs include NP, AF, hyaline CE, and disc perichondrium. At present, stem cell therapy mainly focuses on NP and AF [45–48].

#### 4.2.2. Cluster #1 Adipose-Derived Mesenchymal Stem Cell, ADMSC

ADMSCs are stem cells with pluripotent differentiation potential, which isolated from adipose tissue that is widely and readily available nowadays. It is the current research hotspot of stem cell therapy for degenerative disc diseases (DDD). Studies have shown that compared with bone marrow derived mesenchymal stem cell (BMSCs), ADMSCs have a higher nucleus pulposus-like differentiation capacity. Therefore, ADMSC may be more suitable for the treatment of DDD [49].

Clarke et al. found that the ability of ADMSC to differentiate into nucleus pulposus cell phenotype was strongly enhanced under the induction of TGF-*β*1, GDF5, and GDF6. The cell culture medium level of sulfated glycosaminoglycans and COL II significantly increased [50].

Han et al. performed the analysis of ADMSCs and degenerated nucleus pulposus cells. In vitro co-culture, it not only demonstrated that ADMSCs could promote the repair of degenerated nucleus pulposus cells, but also the first comprehensive identification of degenerative myeloid when co-cultured with ADMSCs nuclear cells were capable of producing lncRNA and mRNA differentially expressed [51]. These research results further provided more valuable information so that people can better understand the role of stem cell therapy in IDD.

The current study shows that ADMSC can be successfully induced to differentiate into nucleus pulposus-like cells under certain conditions to repair the degenerated intervertebral disc, and partially grow biofactors can enhance the repair of ADMSCs. However, the long-term efficacy and safety of ADMSC clinical trials still need to be further verified [52].

#### 4.2.3. Cluster #2 Intervertebral Disc Degeneration, IDD

There are many factors for the degeneration of intervertebral disc, but the most important reason is the decrease of apoptosis and activity of nucleus pulposus cells, which is recognized by the world. The nucleus pulposus cells in the intervertebral disc are in a microenvironment such as hypoxia, acidity, hypertonicity, and lack peripheral blood nutrient supply, which are totally different from other cells in the intervertebral disc [53].

However, the number of NP cells in the intervertebral disc is small, and the rate of cell regeneration is lower than the rate of apoptosis and aging. Under natural conditions, it is difficult for the degenerated intervertebral disc to regenerate and repair to achieve the desired effect. Therefore, it is urgent to find a seed cell that can replace the degenerated NP cells to delay the process of intervertebral disc degeneration. NP cells and chondrocytes are very similar in terms of molecular markers and cell phenotypes, so stem cells that can differentiate into chondrocytes are the best source of cell transplantation for the treatment of intervertebral disc degeneration [54].

Therefore, IDD is difficult to repair by itself and is irreversible. In view of this feature, more and more teams have begun to use stem cells to intervene in the IDD process in order to slow down the IDD process or repair the degenerated intervertebral disc, including mesenchymal stem cells (MSC), intervertebral disc-derived stem cells (IVDSC), and pluripotent stem cells (PSC) [55].

#### 4.2.4. Cluster #3 Degenerative Disc Model

At present, there are more than a dozen animals used to construct intervertebral disc degeneration models, such as mice, rabbits, dogs, pigs, sheep, cattle, and primates. Primates such as monkeys and orangutans are close relatives of humans, and their intervertebral discs are quite similar with human intervertebral discs in terms of physiological structure and biomechanics. However, the current animal experimental research is limited by animal sources, experimental funds, practical operations, and ethics. Therefor primates are rarely used to construct intervertebral disc degeneration models. In addition, large mammals also have disadvantages such as high price and difficulty in feeding [56].

Nowadays, the ideal animal models are small animals such as rabbits and rats. These two animals have the advantages of pure species, many sources, easy to raise, and low price. Although the intervertebral discs of tetrapods lack comparison with human intervertebral discs, they have made great contributions to the pathogenesis and treatment of intervertebral discs [57].

### 4.3. Research Frontiers and Trends on Stem and Progenitor Cells in Intervertebral Discs

The analysis of keyword bursts can grasp the research frontier and latest progress in the field of stem and progenitor cells in intervertebral discs. 4 keywords with the strongest citation bursts appeared from 2017 to 2021, including “microenvironment”, “activation”, “intervertebral disc degeneration”, and “oxidative stress”.

In recent years, related studies have shown that intervertebral disc degeneration is not only affected by the environment and genes, but also related to the microenvironment in intervertebral disc, such as oxygen content, nutrients, and growth factors, which can deteriorate the metabolic environment of nucleus pulposus cells, strengthen anaerobic metabolism, accumulate lactic acid, change acidity, and aggravate intervertebral disc degeneration [58]. Bibby et al. studied the standard unit of lumbar intervertebral disc and found that the glucose concentration and oxygen partial pressure in the endplate area were positively correlated with the cell density in the nucleus pulposus and inversely proportional to the lactate concentration [59], while Mokhbi Soukane studied the standard unit of lumbar intervertebral disc. It was confirmed that the lactate concentration in the blood supply of the endplate cartilage was positively correlated with the degeneration of the intervertebral disc in the corresponding stage [60].

According to the free radical theory of aging, the decline of tissue and organ function is closely related to the oxidative stress induced by reactive oxygen species (ROS) [61]. The occurrence and progression of intervertebral disc degeneration is no exception [62, 63]. In the signaling pathway network of nucleus pulposus cells, ROS acts as an important mediator, regulating extracellular matrix metabolism, pro-inflammatory factor phenotype, apoptosis, autophagy, and aging. On the other hand, the antioxidant proteins in the degenerated intervertebral disc tissue were significantly decreased, which significantly reduced the antioxidant capacity of the intervertebral disc tissue. These changes lead to a redox imbalance in disc cells, which are vulnerable to oxidative damage [64].

The pathophysiological role of oxidative stress on intervertebral disc degeneration is complex. More and more studies are devoted to elucidate the relationship between oxidative stress and intervertebral disc degeneration, and it is found that oxidative stress may be a key factor of intervertebral disc degeneration. Antioxidative stress therapy recognized as a promising treatment for disc degeneration [65]. However, in vitro experiments are insufficient to support the true effectiveness of these antioxidants in preventing or delaying human disc degeneration. Therefore, further clinical research is needed.

## 5. Limitations

Bibliometric analysis is widely used to measure the impact of articles in recent years. However, there are still some limitations. First, we only used the core collection of Web of Science (WOS) for searching literature. The more databases we use, the more information we can get and analyze. Other databases such as InCites and MEDLINE should be considered in future. Second, the main language of WOS is English. Articles written by other languages are excluded, which means some relevant articles to be not included. Third, citation number of each literature is time-dependent. Different time to search the articles, different citations may obtain. However, the trend of citation number of each literature is nearly the same.

This is the first research focusing on stem and progenitor cells in intervertebral disc by an analysis of the scientific landscape using bibliometric method. Our results can benefit scholars involved in the field of intervertebral disc degeneration by better understanding future research directions and trends. They can more specifically improve the state of treatment of intervertebral disc degeneration by paying more attention to adipose-derived mesenchymal stem cell and oxidative stress.

## 6. Conclusion

We demonstrated that research on stem and progenitor cells in intervertebral discs was in a rapid development stage. China had the most publications, and USA played a significant role with highest citations and H-index. The most high-yield author, organization, country, research directions, funds, and journals were Chen QX from Zhejiang University, Zhejiang University, China, Cell Biology, National Natural Science Foundation of China, and *Spine*, respectively. Top 4 research hotspots contained “human intervertebral disc”, “adipose-derived mesenchymal stem cell”, “intervertebral disc degeneration”, and “degenerative disc model”. Meanwhile, research frontiers and trends were “microenvironment”, “activation”, “intervertebral disc degeneration”, and “oxidative stress”.

Our results can benefit researches by quickly grasp research hotspots and trends, which can provide a new perspective for further research.

## Figures and Tables

**Table 1:** Top 20 most cited articles on stem and progenitor cells in intervertebral discs.

First author	Article title	Journal	Publication year	Total citations	Impact factor
Sakai, D	Transplantation of mesenchymal stem cells embedded in Atelocollagen((R)) gel to the intervertebral disc: a potential therapeutic model for disc degeneration	Biomaterials	2003	311	14.593
Sakai, D	Regenerative effects of transplanting mesenchymal stem cells embedded in atelocollagen to the degenerated intervertebral disc	Biomaterials	2006	277	14.593
Sakai, D	Exhaustion of nucleus pulposus progenitor cells with ageing and degeneration of the intervertebral disc	Nature Communications	2012	267	14.919
Sakai, D	Differentiation of mesenchymal stem cells transplanted to a rabbit degenerative disc model - potential and limitations for stem cell therapy in disc regeneration	Spine	2005	265	3.468
Richardson, SM	Mesenchymal stem cells in regenerative medicine: docus on articular cartilage and intervertebral disc regeneration	Methods	2016	251	3.608
Risbud, MV	Differentiation of mesenchymal stem cells towards a nucleus pulposus-like phenotype in vitro: implications for cell-based transplantation therapy	Spine	2004	250	3.468
Crevensten, G	Intervertebral disc cell therapy for regeneration: mesenchymal stem cell implantation in rat intervertebral discs	Annals of Biomedical Engineering	2004	241	3.934
Sakai, D	Stem cell therapy for intervertebral disc regeneration: obstacles and solutions	Nature Reviews Rheumatology	2015	240	20.543
Richardson, SM	Intervertebral disc cell-mediated mesenchymal stem cell differentiation	Stem Cells	2006	227	6.277
Risbud, MV	Evidence for skeletal progenitor cells in the degenerate human intervertebral disc	Spine	2007	213	3.468
Steck, E	Induction of intervertebral disc-like cells from adult mesenchymal stem cells	Stem Cells	2005	202	6.277
Dang, JM	Temperature-responsive hydroxybutyl chitosan for the culture of mesenchymal stem cells and intervertebral disk cells	Biomaterials	2006	190	14.593
Vadala, G	Mesenchymal stem cells injection in degenerated intervertebral disc: cell leakage may induce osteophyte formation	Journal of Tissue Engineering and Regenerative Medicine	2012	187	3.963
Hiyama, A	Transplantation of mesenchymal stem cells in a canine disc degeneration model	Journal of Orthopaedic Research	2008	184	2.359
Minogue, BM	Characterization of the human nucleus pulposus cell phenotype and evaluation of novel marker gene expression to define adult stem cell differentiation	Arthritis and Rheumatism	2010	161	8.955
Arai, F	Mesenchymal stem cells in perichondrium express activated leukocyte cell adhesion molecule and participate in bone marrow formation	Journal of Experimental Medicine	2002	158	14.307
Henriksson, HB	Transplantation of human mesenchymal stems cells into intervertebral discs in a xenogeneic porcine model	Spine	2009	151	3.468
Henriksson, HB	Identification of cell proliferation zones, progenitor cells and a potential stem cell niche in the intervertebral disc region A study in four species	Spine	2009	148	3.468
Richardson, SM	Mesenchymal stem cells in regenerative medicine: opportunities and challenges for articular cartilage and intervertebral disc tissue engineering	Journal of Cellular Physiology	2010	143	6.384
Sobajima, S	Feasibility of a stem cell therapy for intervertebral disc degeneration	Spine Journal	2008	141	4.166

**Table 2:** The top 5 high-yield authors, organizations, and countries on stem and progenitor cells in intervertebral discs.

Category	Rank	Items	Records	H-index	Total citations	Average citations
Author	1	Chen, QX, Zhejiang University	22	15	571	25.95
	1	Zhou, Y, Army Medical University	22	12	544	24.73
	3	Li, FC, Zhejiang University	20	15	527	26.35
	3	Liang, CZ, Zhejiang University	20	15	560	28.00
	5	Li, H, Shanghai Jiao Tong University	19	15	637	33.53

Organization	1	Zhejiang University	33	16	726	22.00
	2	Army Medical University	29	13	609	21.00
	2	League of European Research Universities-LERU	29	18	1382	47.66
	4	Huazhong University of Science & Technology	26	13	419	16.12
	5	University of Hong Kong	18	15	1092	60.67

Country	1	China	231	33	4523	19.58
	2	USA	94	37	4792	50.98
	3	England	32	18	1654	51.69
	4	Japan	30	21	2612	87.07
	5	Switzerland	23	15	950	41.30

**Table 3:** The top 5 high-yield research directions, funds, and journals with the most publications on stem and progenitor cells in intervertebral discs.

Category	Rank	Items	Records	H-index	Total citations	Average citations
Research direction	1	Cell Biology	181	35	4421	24.43
	2	Orthopedics	92	35	4347	47.25
	3	Research & Experimental Medicine	78	24	1997	25.60
	4	Engineering	74	28	3034	41.00
	5	Neurosciences & Neurology	63	33	3335	52.94

Fund	1	National Natural Science Foundation of China (NSFC)	157	27	2798	17.82
	2	National Institutes of Health (NIH) - USA	30	19	1239	41.30
	2	United States Department of Health & Human Services	30	19	1239	41.30
	4	European Commission	18	11	545	30.28
	4	National Key Research and Development Program of China	18	11	345	19.17

Journal	1	Spine	27	20	2025	75.00
	2	Stem Cells International	23	12	359	15.61
	3	Tissue Engineering Part A	16	13	611	38.19
	4	Spine Journal	15	12	669	44.60
	5	Stem Cell Research & Therapy	13	9	348	26.77

**Table 4:** Details of top 9 clusters for researches on stem and progenitor cells in intervertebral discs.

Cluster no.	Size (*n*)	Silhouette	Mean (year)	LSI	LLR	MI
0	65	0.605	2011	Mesenchymal stem cell; intervertebral disc; stem cell; intervertebral disc degeneration; nucleus pulposus | nucleus pulposus cell; disc cell; mesenchymal stem; disc degeneration; disc regeneration	Human intervertebral disc; skeletal progenitor cell; human cartilage endplate; human nucleus; potential stem cell niches	Phenotypic marker; human degenerative intervertebral disc; iron oxide; cell survival; hypoxic-preconditioned bone
1	64	0.665	2012	Mesenchymal stem cell; adipose-derived stem cell; nucleus pulposus; nucleus pulposus cell; intervertebral disc | marrow-derived mesenchymal stem cell; rabbit model; signaling pathway; pulposus-like differentiation; collagen-induced nucleus	Adipose-derived mesenchymal stem cell; adipose-derived stem cell; co-culture system; nucleus pulposus; pulposus-like cell	Phenotypic marker; human degenerative intervertebral disc; iron oxide; cell survival; hypoxic-preconditioned bone
2	56	0.817	2011	Mesenchymal stem cell; intervertebral disc degeneration; nucleus pulposus cell; intervertebral disc; stem cell | nucleus pulposus; human cartilage; progenitor cell; adipose-derived stem cell; pulposus-derived mesenchymal stem cell	Intervertebral disc degeneration; nutrition deficiency; autologous hematopoietic progenitor cell support; high-dose chemotherapy; optic disc	Phenotypic marker; human degenerative intervertebral disc; iron oxide; cell survival; hypoxic-preconditioned bone
3	52	0.747	2009	Mesenchymal stem cell; intervertebral disc; nucleus pulposus cell; human mesenchymal stem cell; degenerative disc model | intervertebral disc degeneration; nucleus pulposus; rabbit model; bone marrow; adipose-derived stem cell	Degenerative disc model; disc regeneration; canine disc degeneration model; disc cell; AKT axis	Phenotypic marker; human degenerative intervertebral disc; iron oxide; cell survival; hypoxic-preconditioned bone
4	49	0.718	2016	Mesenchymal stem cell; intervertebral disc; nucleus pulposus; stem cell; intervertebral disc degeneration | progenitor cell; regenerative medicine; articular cartilage; enhanced regenerative effect; adipose stem	Nucleus pulposus regeneration; annulus fibrosus regeneration; pulposus-based cell delivery system; cyclic compression; perfusion bioreactor	Cell survival; hypoxic-preconditioned bone; phenotypic marker; human degenerative intervertebral disc; iron oxide
5	43	0.735	2014	Mesenchymal stem cell; intervertebral disc; human cartilage; endplate-derived stem cell; splicing event | nucleus pulposus progenitor cell; mesenchymal stem cell differentiation; disc cell; collagen type ii; hypoxic condition	Human cartilage; endplate-derived stem cell; splicing event; genome-wide analysis; stromal cell	Human degenerative intervertebral disc; phenotypic marker; iron oxide; cell survival; hypoxic-preconditioned bone
6	37	0.725	2011	Mesenchymal stem cell; nucleus pulposus cell; human mesenchymal stem cell; vitro study; pulposus-like cell | adipose stem cell; configuration effect; co-cultured stem cell; matrix production; low back pain patient	3d culture; alginate beads hypoxia bone; synthetic peptide b2a; modeling nucleus; vitro study	Phenotypic marker; human degenerative intervertebral disc; iron oxide; cell survival; hypoxic-preconditioned bone
7	37	0.681	2013	Mesenchymal stem cell; nucleus pulposus; scaffold-free cartilage-like disc-shaped cell sheet; shrinkage-free preparation; using human bone marrow | intervertebral disc degeneration; human mesenchymal stem cell; pentosan polysulfate; ovine model; mesenchymal progenitor cell	Shrinkage-free preparation; scaffold-free cartilage-like disc-shaped cell sheet; using human bone marrow; human placenta-derived mesenchymal stem cell; functional regeneration	Phenotypic marker; human degenerative intervertebral disc; iron oxide; cell survival; hypoxic-preconditioned bone
8	27	0.887	2005	Polylactide disc; temporomandibular joint disc; mesenchymal stem cell; intervertebral disc; tissue engineering | nucleus pulposus; bone marrow stem cell; differential response; chitosan hydrogel; iron oxide	Polylactide disc; temporomandibular joint disc; bone marrow formation; tissue engineering; leukocyte cell adhesion molecule	Mesenchymal stem cell; intervertebral disc; nucleus pulposus cell; iron oxide; phenotypic marker

## Data Availability

The authors confirm that all data underlying the findings are fully available upon request.
